# Pharmacokinetic herb-drug interactions: Altered systemic exposure and tissue distribution of ciprofloxacin, a substrate of multiple transporters, after combined treatment with *Polygonum capitatum* Buch.-Ham. ex D. Don extracts

**DOI:** 10.3389/fphar.2022.1033667

**Published:** 2022-10-25

**Authors:** Ziqiang Li, Xi Du, Shuang Tian, Shanshan Fan, Xurui Zuo, Yanfen Li, Ruihua Wang, Baohe Wang, Yuhong Huang

**Affiliations:** ^1^ Second Affiliated Hospital of Tianjin University of Traditional Chinese Medicine, Tianjin, China; ^2^ Graduate School, Tianjin University of Traditional Chinese Medicine, Tianjin, China

**Keywords:** herb-drug interaction (HDI), *Polygonum capitatum* extract, drug-drug interaction (DDI), ciprofloxacin (CIP), drug transporters, tissue distribution

## Abstract

**Background:** Combination of *Polygonum capitatum* Buch.-Ham. ex D. Don extract (PCE) and ciprofloxacin (CIP) was commonly prescribed in the treatment of urinary tract infections. Their pharmacokinetic herb-drug interactions (HDIs) were focused in this study to assess potential impact on the safety and effectiveness.

**Methods:** A randomized, three-period, crossover trial was designed to study the pharmacokinetic HDI between PCE and CIP in healthy humans. Their pharmacokinetic- and tissue distribution-based HDIs were also evaluated in rats. Gallic acid (GA) and protocatechuic acid (PCA) were chosen as PK-markers of PCE in humans and rats. Potential drug interaction mechanisms were revealed by assessing the effects of PCE on the activity and expression of multiple transporters, including OAT1/3, OCT2, MDR1, and BCRP.

**Results:** Concurrent use of PCE substantially reduced circulating CIP (approximately 40%–50%) in humans and rats, while CIP hardly changed circulating GA and PCA. PCE significantly increased the tissue distribution of CIP in the prostate and testis of rats, but decreased in liver and lungs. Meanwhile, CIP significantly increased the tissue distribution of GA or PCA in the prostate and testis of rats, but decreased in kidney and heart. In the transporter-mediated *in vitro* HDI, GA and PCA presented inhibitory effects on OAT1/3 and inductive effects on MDR1 and BCRP.

**Conclusion:** Multiple transporter-mediated HDI contributes to effects of PCE on the reduced systemic exposure and altered tissue distribution of CIP. More attention should be paid on the potential for PCE-perpetrated interactions.

## 1 Introduction

Communicable diseases are still leading causes of death and disability globally, according to WHO’s Global Health Estimates 2000–2019 (GBD Diseases and Injuries, 2020). Antibiotics present a vital role in universal health insurance and global health protection. However, the clinical pipeline and recently approved antibiotics are insufficient to tackle the challenge of increasing emergence and spread of antimicrobial resistance. In recent years, traditional Chinese medicine (TCM)-based herbal therapies have been widely used to treat infectious diseases and solve the problem of microbial resistance. In particular, since the outbreak of COVID-19, TCM has fully participated in the prevention, control and treatment of the epidemic and made important contributions (Huang et al., 2021). According to the WHO Expert Meeting on Evaluation of TCM in the Treatment of COVID-19, WHO encouraged Member States to consider the integrated traditional Chinese and Western medicine model (WHO, 2022).

*Polygonum capitatum* Buch.-Ham. ex D. Don (*P. capitatum*), a Chinese herbal plant, is often used alone or in combination with antibacterial agents to treat urinary tract infections, pyelonephritis, and prostatitis in China (Chinese Pharmacopoeia Commission, 2010; Liao et al., 2011). Relinqing^®^ granule, a *P. capitatum*-based Chinese patent medicine, has been approved by the National Medical Products Adminnistration (NMPA) and officially listed in the Chinese Pharmacopoeia since 2010 (Chinese Pharmacopoeia Commission, 2010). A systematic review of randomized controlled trials indicated that the combination of Relinqing^®^ with antibiotics can improve the total effective rate of urinary tract infections in comparison with the antibiotic therapy alone (Pu et al., 2016). Evidence-based herb-drug interactions (HDI) studies are expected to become a necessary evaluation for the rational use of *P. capitatum*-based product with other drugs prescribed for the same indications. Therefore, we focused on pharmacokinetic- and tissue distribution-based HDIs between *P. capitatum* extracts (PCE) and fluoroquinolone antibacterial agents in this study.

Gallic acid (GA) and protocatechuic acid (PCA) were identified as appropriate pharmacokinetic markers (PK-markers) of *P. capitatum* because of their extensive pharmacological activities, high systemic exposures, and acceptable pharmacokinetic properties. Specifically, they are the two most abundant phenolic acids in *P. capitatum* (Liao et al., 2013; Zhang et al., 2013a; Zhang et al., 2013b; Li et al., 2021b), and their anti-microbial, anti-inflammatory, anti-oxidant, and analgesic activities associated with *P. capitatum* efficacy (Liao et al., 2011; Khan et al., 2015; Choubey et al., 2018; Song et al., 2020; Bai et al., 2021). The qualitative and quantitative analysis of PCE systemic exposure showed that GA and PCA possess a relatively high exposure in rats (Ma et al., 2015, 2016; Huang et al., 2019; Guan et al., 2022) and humans (Li et al., 2021b). After oral administration of PCE, GA and PCA underwent a rapid absorption (Ma et al., 2015; Li et al., 2021b), a dose-dependent profile (Ma et al., 2015), and a relatively targeted distribution in kidney tissue (Ma et al., 2016). Consequently, GA and PCA were chosen as PCE tracer components in the pharmacokinetic- and tissue distribution-based HDIs studies.

Ciprofloxacin (CIP) was selected as a representative fluoroquinolone agent in this study because 1) it is a commonly used antibiotic in the treatment of urinary system diseases alone or in combination (Bonkat et al., 2022), and 2) it is cleared by active tubular secretion and intestinal excretion (Höffken et al., 1985; Rohwedder et al., 1990; Vance-Bryan et al., 1990). CIP’s absolute bioavailability is approximately 70%, with no substantial loss by first pass metabolism, and its metabolites together account for approximately 10% of an oral dose (Vance-Bryan et al., 1990). Renal clearance of CIP accounts for 2/3 of its total clearance and exceeds the normal glomerular filtration rate, suggesting that active tubular secretion may play a role (Höffken et al., 1985; Mulgaonkar et al., 2012). Approximately 60% of CIP is excreted in unchanged form into the urine (Vance-Bryan et al., 1990). Approximately 20% of an intravenous dose of CIP is eliminated into the intestine (Rohwedder et al., 1990; Haslam et al., 2011). At physiological pH = 7.4, CIP predominantly exists as a zwitterion indicating that both anion and cation transporters may contribute to its excretion (Vanwert et al., 2008; Haslam et al., 2011; Arakawa et al., 2012; Mulgaonkar et al., 2012). CIP is a known substrate of the ATP-binding cassette transporters, which have been implicated in its intestinal secretion, biliary excretion and secretion into breast milk (Vance-Bryan et al., 1990; Merino et al., 2006; Ando et al., 2007; Mulgaonkar et al., 2012). Overall, drug transporter-mediated HDIs were assessed to reveal the altered systemic exposure and tissue distribution of CIP after combined treatment with PCE in this study.

## 2 Materials and methods

### 2.1 Chemicals and reagents

Relinqing^®^ granules (4 g, 170207) and *P. capitatum* extract (PCE, 19338D) were kindly provided by Guizhou Warmen Pharmaceutical Co., Ltd. (Guiyang, China). Ciprofloxacin hydrochloride tablets (0.25 g, 150202) and ciprofloxacin lactate and sodium chloride injection (Ciprobay^®^, 100 ml:0.2 g ciprofloxacin and 0.9 g sodium chloride, BXHK JX1) were commercially obtained from PKU HealthCare Corp., Ltd. (Chongqing, China) and Bayer Pharma AG (Leverkusen, Germany), respectively. Reference standards of GA (110831–201906), PCA (110809–201906), CIP (130451–201904), chloromycetin (130555–201704), and ofloxacin (130454–202007) were purchased from National Institute for Food and Drug Control (Beijing, China). Dulbecco’s modified Eagle’s medium (DMEM), nonessential amino acids solution (NEAA), fetal bovine serum (FBS), and penicillin-streptomycin solution were purchased from Invitrogen (Grand Island, NY, United States). BCA Protein Assay Kit (P0011) and complete Freund’s adjuvant (CFA, P2036-10 ml) were provided by Beyotime Biotechnology (Shanghai, China). Isoflurane (S10010533) was obtained from Shanghai Yuyan Instrument Co., Ltd. (Shanghai, China). Acetonitrile, methanol and formic acid were purchased from Thermo Fisher Scientific (Waltham, MA). Other reagents were commercially available and of analytical grade.

### 2.2 Pharmacokinetic herb-drug interactions in healthy volunteers

#### 2.2.1 Healthy subjects

Twelve healthy male volunteers aged 18–35 years who weighed at least 50 kg and had a body mass index of 19–24 kg/m^2^ were eligible for recruitment. Additional inclusion criteria included a healthy status confirmed by a review of the medical history, a physical examination, clinical laboratory tests, and a non-smoking status. Subjects were excluded if they had any allergies, hematological abnormalities, or a history of renal, hepatic, or gastrointestinal diseases. Subjects were also excluded if they recently had taken any medications or ingested grapefruit juice, St. John’s wort, or agents that interact with either *P. capitatum* or CIP for at least 2 weeks prior to dosing and during the study. Safety was monitored by performing clinical laboratory tests, 12-lead ECGs, recordings of vital signs, and physical examinations at baseline and scheduled times. The protocol was approved by the Ethics Committee of the Second Affiliated Hospital of Tianjin University of Traditional Chinese Medicine (Ethical Approval No: 2015-033-03). All subjects provided written informed consent prior to enrollment.

#### 2.2.2 Study design in healthy volunteers

**FIGURE 1:**
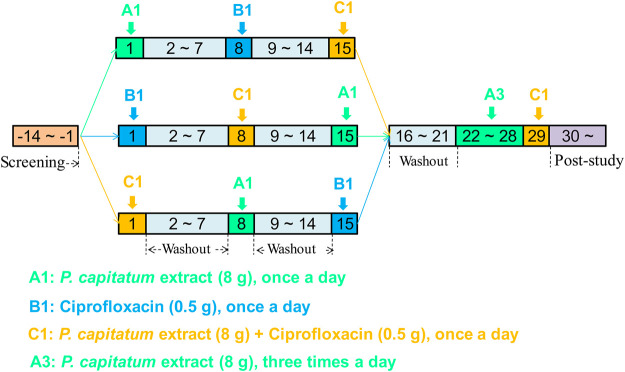
Schematic illustration of the study design in healthy subjects.

A randomized, three-period, crossover trial was designed to study the herb-drug interactions in healthy male subjects following the administration of single-dose treatments with PCE (8 g Relinqing^®^), CIP (0.5 g), or PCE (8 g) + CIP (0.5 g) (Registration No: ChiCTR-OPh-16010029). Each treatment period was separated by a washout period of 7 days. Subjects received multiple doses of PCE from day 22 to day 28 and a single dose of the combination of PCE and CIP treatment on day 29 (Figure 1). Subjects were offered standard meals 4 and 10 h after dosing. Water was not permitted during the hour before and the hour after dosing, with the exception of 240 ml administered with dosing; additional water intake was allowed at all other times. On days 1, 8, 15 and 29, 3 ml blood samples were collected from the antecubital vein catheter prior to drug administration and at 0.08, 0.17, 0.33, 0.5, 0.75, 1, 1.5, 2, 3, 4, 6, 8, 12, and 24 h after dosing. All samples were stored at −80°C until analysis.

### 2.3 Pharmacokinetic- and tissue distribution-based herb-drug interaction studies in rats

#### 2.3.1 Experimental animals

Male Sprague-Dawley rats weighing 200–220 g were purchased from HFK Bioscience Co., Ltd. (SCXK 2019–0008, Beijing, China). All of the experimental procedures were carried out according to the Guidance for Ethical Treatment of Laboratory Animals. Approval for this study was granted by the Institutional Animal Care and Use Committee of Tianjin University of Traditional Chinese Medicine. All animals were housed at the individually ventilated cages (three rats per cage) in a temperature-controlled room under a 12-h light/dark cycle. Water and food were supplied *ad libitum* and the rats were fasted only with free access to water for 12 h prior to experiment.

#### 2.3.2 Pharmacokinetic-based herb-drug interaction study in rats

In the pharmacokinetic-HDI study, eighteen rats were randomly assigned to three experimental groups (*n* = 6 per group). Rats in the PCE, CIP, and PCE + CIP groups were orally given a single dose of PCE (0.72 g/kg, comparable to a clinical dose of 8 g Relinqing^®^), CIP (0.045 g/kg, comparable to a clinical dose of 0.5 g ciprofloxacin hydrochloride tablets), PCE (0.72 g/kg) and CIP (0.045 g/kg), respectively. Blood samples (approx. 200 μl) were collected into heparinized tubes prior to dosing and at 0.17, 0.5, 1, 1.5, 2, 2.5, 3, 4, 6, 8, 12, and 24 h after oral dosing. Rats were euthanized with isoflurane at the end of the experiment. Plasma samples were separated after centrifugation at 3,000 rpm for 10 min and stored at −80°C until analysis.

#### 2.3.3 Tissue distribution-based herb-drug interaction study in rats

In the tissue distribution-HDI study, thirty-six rats were randomly assigned to three experimental groups (*n* = 12 per group). Rats in the PCE, CIP, and PCE + CIP groups were given a single dose of PCE (0.72 g/kg, comparable to a clinical dose of 8 g Relinqing^®^, *i.g.*), CIP (0.036 g/kg, comparable to a clinical dose of 0.4 g Ciprobay^®^, *i.v.*), PCE (0.72 g/kg) and CIP (0.036 g/kg), respectively. Three rats in each group were randomly euthanized with isoflurane at 0.5, 1, 3, and 5 h, respectively. The heart was perfused with normal saline to remove blood from the tissues. The heart, liver, lung, kidney, prostate, testis, seminal vesicle gland (SVG), and spleen were collected and then blotted on filter paper. Plasma and tissue samples were stored at −80°C until analysis.

### 2.4 Effect of PCE on the transport of ciprofloxacin mediated by multiple transporters

#### 2.4.1 Inhibitory effects of gallic acid and protocatechuic acid on the activity of multiple transporters

Considering the relative exposure characteristics, GA and PCA were selected as PK-marker and Q-marker components of PCE in previous studies (Ma et al., 2015; Ma et al., 2016; Li et al., 2021b). In this study, we investigated the concentration-dependent inhibitions of GA and PCA on multiple transporters, including OAT1, OAT3, OCT2, MDR1, and BCRP. Stably expressing cell lines of hOAT1-MDCK, hOAT3-MDCK, hOCT2-S2, hMDR1-MDCK, hBCRP-MDCK, and their mock cells were obtained from Japan Fuji Biomedical Co., Ltd. The cells were maintained in DMEM (Sigma) supplemented with 10% FCS, 1% non-essential amino acids (Sigma), and 2% L-glutamine. The medium for the Transwell^®^ plates (Corning Costar, Cambridge, MA, United States) was supplemented with 0.1% gentamicin. Membrane filters were placed in a 12 mm well with 1.5 and 0.5 ml of culture medium in the basolateral and apical compartments, respectively. Cells were cultured at 37°C in an atmosphere of 5% CO_2_. The inhibition experiments were adapted from previous methods with minor modifications (Li et al., 2021a; 2021b).

Briefly, the hOAT1-MDCK, hOAT3-MDCK, and hOCT2-S2 cells were trypsinized and suspended in the culture medium to provide a density of 1.5 × 10^5^ cells/ml. After incubating for 48 h, the cells were washed twice with preheated DPBS and then pre-incubated with DPBS for 10 min at 37°C. 500 µl of DPBS containing probe substrates was added to initiate the uptake in the presence or absence of GA, PCA or positive inhibitors. The hMDR1-MDCK and hBCRP-MDCK cells were suspended at a density of 4.0 × 10^5^ cells/ml. After cultivation for 7 days, cells were washed twice and equilibrated for 30 min at 37°C with the pre-warmed HBSS buffer. 1.5 ml of transport buffer containing probe substrates was added in the basolateral compartment to initiate the efflux in the presence or absence of GA, PCA or positive inhibitors. The designated GA or PCA concentrations were 0.3, 1, 3, 10, 30 μM in the incubation system. ^14^C-PAH (5 µM), ^3^H-ES (0.05 µM), ^14^C-TEA (5 µM), rhodamine 123 (10 µM), and lucifer yellow (10 µM) were chosen as probe substrates of hOAT1, hOAT3, hOCT2, hMDR1, and hBCRP, respectively. Probenecid (100 µM), cimetidine (600 µM), probenecid (100 µM), verapamil (10 µM), and cyclosporine (20 µM) were selected as positive inhibitors of hOAT1, hOAT3, hOCT2, hMDR1, and hBCRP, respectively. The content of DMSO was below 1% and constant in all inhibition experiments. All experiments were performed in triplicate. The cell lysate and 3.0 ml of aquasol-2 scintillation fluid were put into a scintillation flask. The radioactive intensity of ^14^C-PAH, ^3^H-ES, and ^14^C-TEA was measured using a Tri-Carb 2910TR scintillator (PerkinElmer, United States). Fluorescence was measured at 485 nm (excitation) and 546 nm (emission) for rhodamine 123, and 425 nm (excitation) and 528 nm (emission) for lucifer yellow. IC_50_ values were determined by employing GraphPad Prism 9.

#### 2.4.2 Effects of PCE on the Bi-directional transport of ciprofloxacin in Caco-2 cells

A bidirectional assay in Caco-2 cells is a preferred method to determine whether an investigational drug is a substrate for P-gp/BCRP or whether an investigational drug is an inhibitor of P-gp/BCRP (US FDA, 2020). The Caco-2 cell lines were obtained from the National Collection of Authenticated Cell Cultures (NCACC, Shanghai, China). The bi-directional transport assay was performed on 12-mm Transwell Permeable Supports with 0.4 mm-pore polycarbonate membrane insert and 1.12 cm^2^ growth areas. Caco-2 cells were maintained in DMEM complete high-glucose medium with L-glutamine, supplemented with 10% FBS and 1% NEAA. The cells were seeded at a density of 3×10^5^ cells/well, incubated at 37°C and 5% CO_2_, and cultured for 21 days with replacement of cell culture medium supplemented with 1% penicillin-streptomycin every second day. Membrane filters were placed in a 12 mm well with 1.5 and 0.5 ml of culture medium in the basolateral and apical compartments, respectively. TEER was measured as an indication of an intact monolayer using a Millicell ERS voltohmmeter (Millipore, Merck). After washing the monolayers with prewarmed PBS, the cells were preincubated with DMEM with and without the investigated drugs, including GA (5, 50 μM), PCA (5, 50 μM) and PCE (10, 100 mg/ml). The transport assays were initiated by the addition of CIP (25 μM) with or without the investigated drugs into the apical or basal compartment. Aliquots of 50 μl were collected from the acceptor compartment at 0.5 h. The apparent permeability coefficient (*P*_app_) was calculated using the following formula: *P*_app_ = (*dC*/*dt*) × *V*_r_/(*A* × *C*_0_), where *dC*/*dt* is the alteration in concentration over time, *V*_r_ is the volume of the receiver compartment, *A* is the area of the cell monolayer, and *C*_0_ is the initial concentration in the donor compartment. The permeation of drug from the apical (A) to the basolateral (B) side of the cells (*P*_app,A-B_) is compared with the permeation in the opposite direction (*P*_app,B-A_). If the net flux ratio is >2, this suggests a probable P-gp or BCRP substrate. If the net flux ratio decreases with increasing concentrations of the investigational drug, this suggests a probable P-gp or BCRP inhibitor (US FDA, 2020).

#### 2.4.3 Effects of PCE on the MDR1 and BCRP mRNA expressions in Caco-2 cells

Caco-2 cells induced with the positive control or tested drugs were then incubated in a 37°C incubator with 5% CO_2_ for 48 h. Bosentan served as a positive control for the induction of MDR1 and BCRP (van Giersbergen et al., 2002). After the incubation period, cells were harvested from the Transwell inserts for RNA extraction. The mRNA expressions of MDR1 and BCRP in cells were detected at 48 h after pretreatments with bosentan (20 μM), GA (50 μM), PCA (50 μM), and PCE (100 mg/ml). Real-time quantitative PCR (qRT-PCR) was performed on an ABI Prism 7900HT Sequence Detection System (Applied Biosystems, Foster City, CA, United States) for the analysis of MDR1 and BCRP genes. Primers of housekeeping gene β-actin was used as internal controls. The primer sequences used for MDR1 amplification were CGC​ACC​TGC​ATT​GTG​ATT​GC (forward) and AGA​TGC​CTT​TCT​GTG​CCA​GC (reverse). The primer sequences used for BCRP amplification were AGC​AGC​AGG​TCA​GAG​TGT​GG (forward) and CTG​AAG​CCA​TGA​CAG​CCA​AG (reverse). The relative gene expression levels were determined using the comparative CT method (ΔΔCT method). The cut-off CT was set at 35 cycles for all analyses. An arbitrary classification system was assigned to the data, designating relative expression levels >2 as high mRNA expression, levels between 2 and 1 as moderate mRNA expression, levels between 1 and 0.1 as low mRNA expression and levels <0.1 as unexpressed.

### 2.5 LC-MS/MS assay for PCE and ciprofloxacin

#### 2.5.1 Sample preparation

The tissue samples were homogenized using a tissue homogenizer (JXFSTPRP-24, Shanghaijingxin Experimental Technology, Shanghai, China) in physiological saline solution (1:4, *w*/*v*). The analytes and internal standards were extracted from tissue homogenates and plasma samples by a simple protein precipitation method. Briefly, an aliquot of 100 μl biological samples, 5 μl internal standard solutions, and 300 μl acetonitrile were vortex-mixed for 3 min and then centrifuged at 14,000 rpm for 10 min. The supernatant was transferred and dried using a gentle stream of nitrogen at 35°C. The residues were reconstituted with 100 μl of 20% acetonitrile solution, vortex-mixed for 1 min, and centrifugated at 14,000 rpm for 10 min. The supernate was utilized for the quantification of GA, PCA, and CIP.

#### 2.5.2 LC-MS/MS conditions

Samples were analyzed using a Waters ACQUITY™ ultra performance liquid chromatography system (Waters Corp., Milford, MA, United States) equipped with an QTRAP5500 triple-quadrupole mass spectrometer (SCIEX, Framingham, MA, United States) and an electrospray source. Data acquisition was controlled by Analyst 1.7.2 software (SCIEX, Concord, ON, Canada).

Chromatographic separation of GA and PCA was achieved on an ACQUITY UPLC BEH C18 (2.1 mm × 100 mm, 1.7 µm; Waters Corp., Milford, MA, United States) with a mobile phase of (A) 0.1% formic acid aqueous solution and (B) 0.1% formic acid acetonitrile at a flow rate of 0.3 ml/min. The gradient elution program was as follows: 0–8.5 min, 97% A; 8.7–11 min, 60% A; 11.5–13.5 min, 10% A; 14–15 min, 97% A. The quantitation of GA and PCA was performed using multiple reaction monitoring (MRM) mode with an electron spray ionization (ESI) source in negative-ionization mode. The source operation parameters were optimized as follows: ion spray voltage, −4,500 V; source temperature, 550°C; ion source gas1, 55 psi; ion source gas2, 60 psi; curtain gas, 35 psi. The precursor-product ion transitions of GA, PCA, and chloramphenicol (internal standard) were 169.0→125.1, 153.1→109.0, and 321.1→152.1, respectively.

The separation of CIP was performed on the same column and mobile phase as GA and PCA. The gradient elution program was as follows: 0–2.5 min, 85% A; 3–4 min, 10% A; 4.5–6 min, 85% A; 14–15 min, 97% A. The quantitation of CIP was performed using MRM mode with an ESI source in positive-ionization mode. The source parameters were chosen as follows: ion spray voltage, 5,500 V; source temperature, 450°C; ion source gas1, 50 psi; ion source gas2, 50 psi; curtain gas, 35 psi. The precursor-product ion transitions of CIP and ofloxacin (internal standard) were 332.1→288.1 and 362.2→318.1, respectively.

### 2.6 Pharmacokinetic analysis

PK parameters were estimated with noncompartmental methods using WinNonlin version 6.4 (Certara, Princeton, NJ). The peak plasma concentration (*C*_max_) and time to reach the peak plasma concentration (*T*_max_) were calculated from the actual plasma concentration data. The area under the plasma concentration-time curve from zero to the last measurable concentration (*AUC*_0-t_) was calculated *via* the linear trapezoidal rule. The *AUC*_0-∞_ was calculated using the following formula: *AUC*_0-∞_ = *AUC*_0-t_ + *C*_t_/*k*_e_, where *C*_t_ is the last plasma concentration measured. The terminal elimination half-life (*t*_1/2_) was calculated as 0.693/*k*_e_. The oral clearance (*CL*/*F*) and volume of distribution (*V*_z_/*F*) were defined as *Dose*/*AUC*_0-∞_ and *Dose*/(*k*_e_·*AUC*_0-∞_), respectively.

### 2.7 Data analysis

All data were summarized as mean ± standard deviation (SD). All statistical analyses were performed using SPSS software (Chicago, IL, United States). Comparisons between groups were performed using unpaired Student’s *t*-test. A *p*-value of less than 0.05 was considered statistically significant. Pharmacokinetic interactions were reported as 90% confidence intervals (CI) for the geometric mean ratios (GMR) of the observed pharmacokinetic measures in the presence and absence of the interacting drug. If the 90% CI for systemic exposure ratios was entirely encompassed within the equivalence range of 0.80–1.25, we concluded that clinically significant difference was not present.

## 3 Results

### 3.1 Pharmacokinetic herb-drug interaction between PCE and ciprofloxacin in healthy volunteers

All recruited subjects were healthy Chinese natives. All enrolled subjects completed the study protocol as planned. Their age, height, weight, and body mass index were summarized in Supplementary Table S1. No major protocol deviations were identified, and no serious adverse reactions were observed throughout the study.

#### 3.1.1 Effect of PCE on the pharmacokinetics of ciprofloxacin in human

**FIGURE 2:**
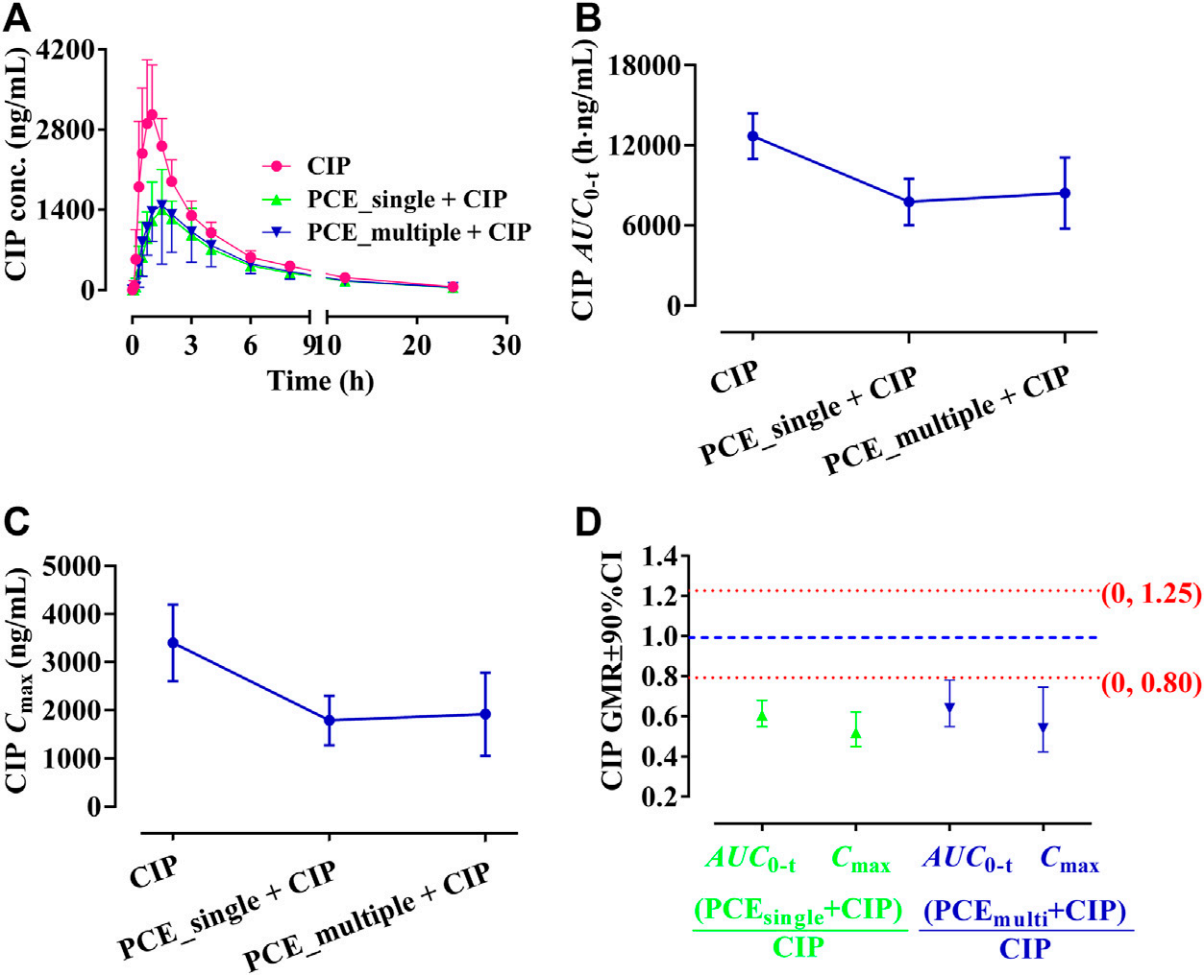
Plasma ciprofloxacin (CIP) concentration-time profiles **(A)**, the systemic exposure parameters includi*ng AUC*_*0-t*_ **(B)** *and C*_max_ **(C)**, *a*nd their 90% CIs for the geometric mean ratios [GMR, **(D)**] in the presence and absence of the single-dose/multiple-dose PCE to healthy humans (mean ± SD, *n* = 12).

The effects of PCE on CIP plasma concentrations were investigated by comparing alterations in PK exposure measures between control and treatment groups. The CIP concentration-time profiles and PK parameters are shown in Figure 2A and Table 1, respectively. The plasma ciprofloxacin *AUC*_0-t_ and *C*_max_ were significantly decreased in the presence of single-dose/multiple-dose PCE (Figures 2B,C). Following combination therapy with a single dose of PCE, the GMRs±90% CI of ciprofloxacin *AUC*_0-t_ and *C*_max_ were 0.61 (0.55, 0.68) and 0.52 (0.45, 0.62), respectively. After pretreatment with multiple doses of PCE, the GMRs ± 90% CI of ciprofloxacin *AUC*_0-t_ and *C*_max_ were 0.64 (0.54, 0.78) and 0.54 (0.42, 0.74), respectively, falling out of the equivalence range of 0.80–1.25 (Figure 2D). The results indicated that the co-administration of PCE significantly reduces the systemic exposure of CIP in human.

**TABLE 1:** Pharmacokinetic parameters of ciprofloxacin after the oral administration of ciprofloxacin hydrochloride tablets (CIP, 0.5 g) with and without PCE (8 g) to human subjects (mean ± SD, *n* = 12).

Parameters	CIP	CIP + PCE_single_ ^b^	CIP + PCE_multiple_ ^c^
*C*_max_ (ng/ml)	3400 ± 796	1920 ± 863*	1790 ± 510*
*AUC*_0-t_ (h·ng/mL)	12700 ± 1690	8410 ± 2640*	7770 ± 1730*
*AUC*_0-∞_ (h·ng/mL)	13200 ± 1800	8790 ± 2690*	8110 ± 1770*
*t*_max_ (h)^a^	1.0 (0.33–1.5)	1.25 (0.5–3)	1.5 (0.5–3)
*k*_e_ (h^−1^)	0.12 ± 0.01	0.12 ± 0.01	0.12 ± 0.01
*t*_1/2_ (h)	5.64 ± 0.49	5.79 ± 0.50	5.66 ± 0.40
*V*_z_/F (L)	313 ± 35.9	524 ± 183*	525 ± 110*
*CL*/F (L/h)	0.64 ± 0.08	1.03 ± 0.31*	1.07 ± 0.21*

^a^ Median (range).

^b^ PCE_single_, a single dose of PCE (8 g).

^c^ Multiple doses of PCE (8 g, TID) for seven consecutive days. *p< 0.05, significant differences were observed when compared with the CIP, group.

#### 3.1.2 Effect of ciprofloxacin on the pharmacokinetics of PCE PK-markers in human

**FIGURE 3:**
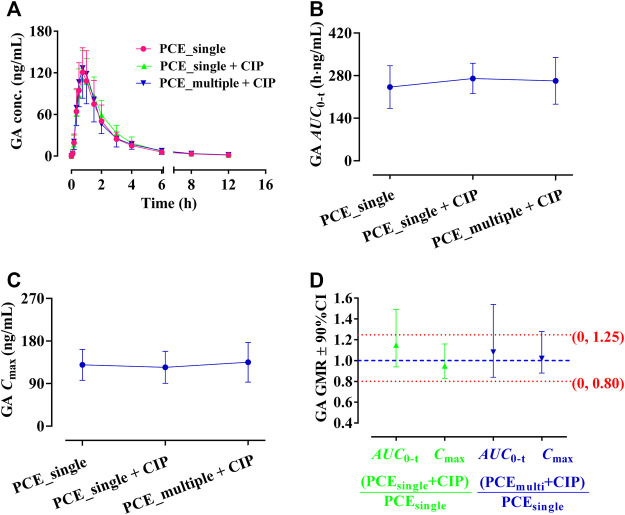
Plasma gallic acid (GA) concentration-time profiles **(A)**, the systemic exposure parameters of GA including *AUC*_0-t_ **(B)** and *C*_max_ **(C)**, and their 90% CIs for the geometric mean ratios [GMR, **(D)**] in the presence and absence of oral ciprofloxacin hydrochloride tablets (CIP) to healthy humans (mean ± SD, *n* = 12).

**FIGURE 4:**
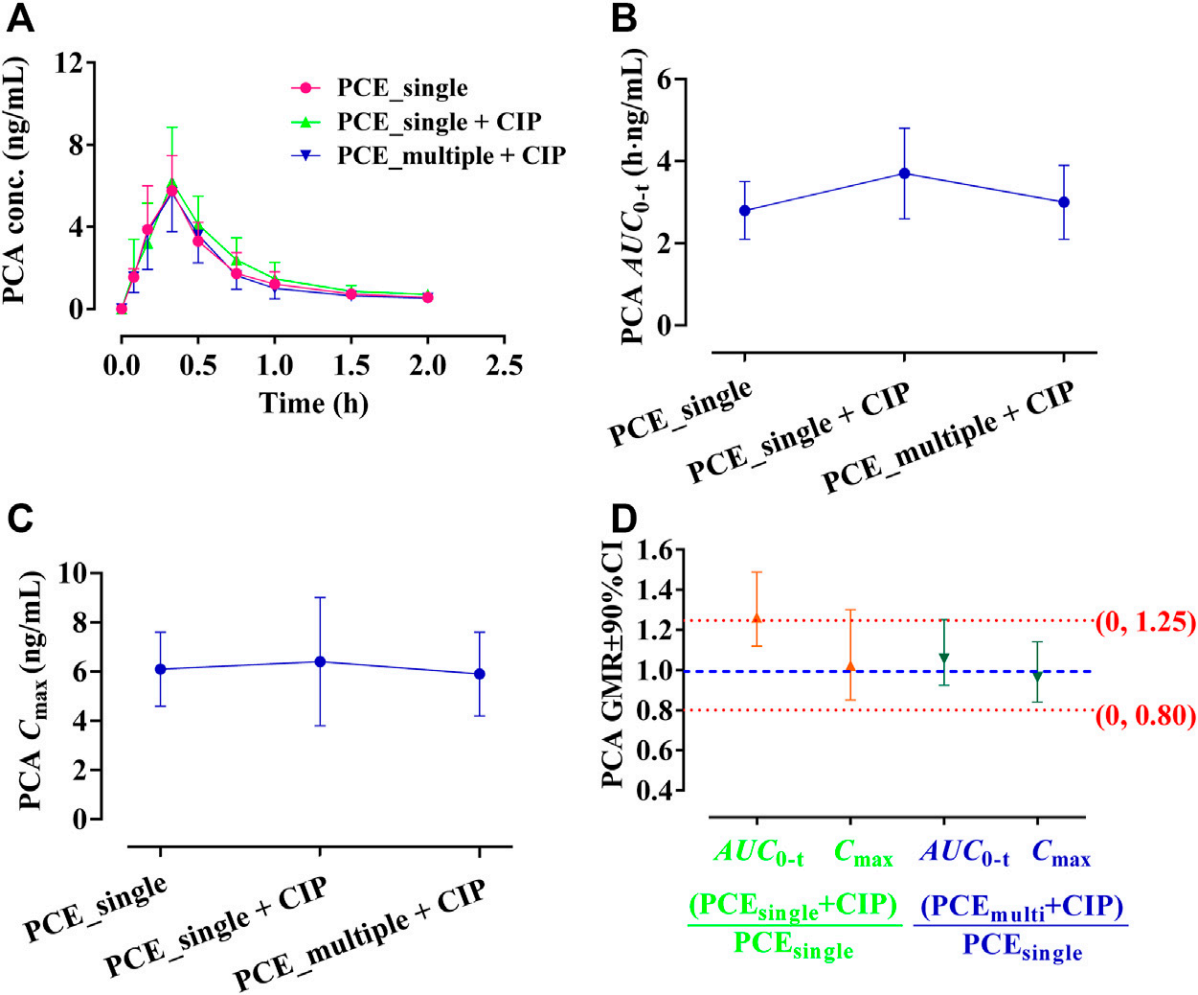
Plasma protocatechuic acid (PCA) concentration-time profiles **(A)**, the systemic exposure parameters of PCA including *AUC*_0-t_ **(B)** and *C*_max_ **(C)**, and their 90% CIs for the geometric mean ratios [GMR, **(D)**] in the presence and absence of oral ciprofloxacin hydrochloride tablets (CIP) to healthy humans (mean ± SD, *n* = 12).

GA and PCA were identified as PK-markers of PCE to present the effects of CIP on PCE systemic exposures. After co-administration of CIP and PCE, the PK changes of GA and PCA are displayed in Figures 3, 4, respectively. Their PK parameters (Table 2) were calculated from the plasma concentration-time curves (Figures 3A, 4A). No significant differences between control and combination treatment groups were observed on the systemic exposure parameters of GA (Figures 3B,C) and PCA (Figures 4B,C). Following co-treatment with CIP and a single dose of PCE, the GMRs ±90% CI of GA *AUC*_0-t_ and *C*_max_ were 1.15 (0.94, 1.49) and 0.95 (0.83, 1.16), respectively (Figure 3D), and the GMRs ± 90% CI of PCA *AUC*_0-t_ and *C*_max_ were 1.26 (1.12, 1.49) and 1.02 (0.85, 1.30), respectively (Figure 4D). Following co-treatment with ciprofloxacin and multiple doses of PCE, the GMRs ± 90% CI of GA *AUC*_0-t_ and *C*_max_ were 1.08 (0.84, 1.54) and 1.02 (0.88, 128), respectively (Figure 3D), and the GMRs±90% CI of PCA *AUC*_0-t_ and *C*_max_ were 1.06 (0.92, 1.25) and 0.96 (0.84, 1.14), respectively (Figure 4D). The results indicated that the co-treatment with CIP does not significantly alter the PK behaviors of PCE PK-markers in humans.

**TABLE 2:** Pharmacokinetic parameters of gallic acid (GA) and protocatechuic acid (PCA) after the oral administration of PCE (8 g) with and without ciprofloxacin hydrochloride tablets (CIP, 0.5 g) to human subjects (mean ± SD, *n* = 12).

Parameters	GA			PCA		
	PCE_single_ ^b^	PCE_single_ + CIP	PCE_multiple_ ^c^+ CIP	PCE_single_	PCE_single_ + CIP	PCE_multiple_+ CIP
*C*_max_ (ng/ml)	130 ± 32.7	124 ± 33.8	135 ± 41.9	6.07 ± 1.48	6.44 ± 2.57	5.88 ± 1.66
*AUC*_0-t_ (h·ng/mL)	242 ± 70.0	271 ± 50.0	263 ± 76.7	2.84 ± 0.72	3.68 ± 1.15	3.03 ± 0.9
*AUC*_0-∞_ (h·ng/mL)	248 ± 67.0	274 ± 50.8	268 ± 77.3	3.39 ± 0.94	4.40 ± 0.93	3.35 ± 0.99
*t*_max_ (h)^a^	0.88 (0.5, 1)	1 (0.5, 1.5)	0.75 (0.5, 1.5)	0.33 (0.17, 0.75)	0.33 (0.33, 0.5)	0.33 (0.17, 0.75)
*k*_e_ (h^−1^)	0.51 ± 0.19	0.47 ± 0.14	0.42 ± 0.11	2.36 ± 0.82	2.05 ± 1.02	2.51 ± 0.85
*t*_1/2_ (h)	1.64 ± 0.92	1.59 ± 0.50	1.78 ± 0.55	0.32 ± 0.11	0.42 ± 0.19	0.31 ± 0.14
*V*_z_/F (L)	322 ± 295	245 ± 71.8	286 ± 75.2	148 ± 23.9	149 ± 65.6	150 ± 54.4
*CL*/F (L/h)	2.10 ± 0.64	1.98 ± 0.71	1.82 ± 0.35	5.64 ± 1.45	4.26 ± 0.91	5.78 ± 1.57

^a^ Median (range).

^b^ PCE_single_, a single dose of PCE (8 g).

^c^ Multiple doses of PCE (8 g, TID) for seven consecutive days. No significant differences were observed when compared with the PCE_single_ group (*p*> 0.05).

### 3.2 Pharmacokinetic herb-drug interaction between PCE and ciprofloxacin in rats

#### 3.2.1 Effect of PCE on the pharmacokinetics of ciprofloxacin in rats

**FIGURE 5:**
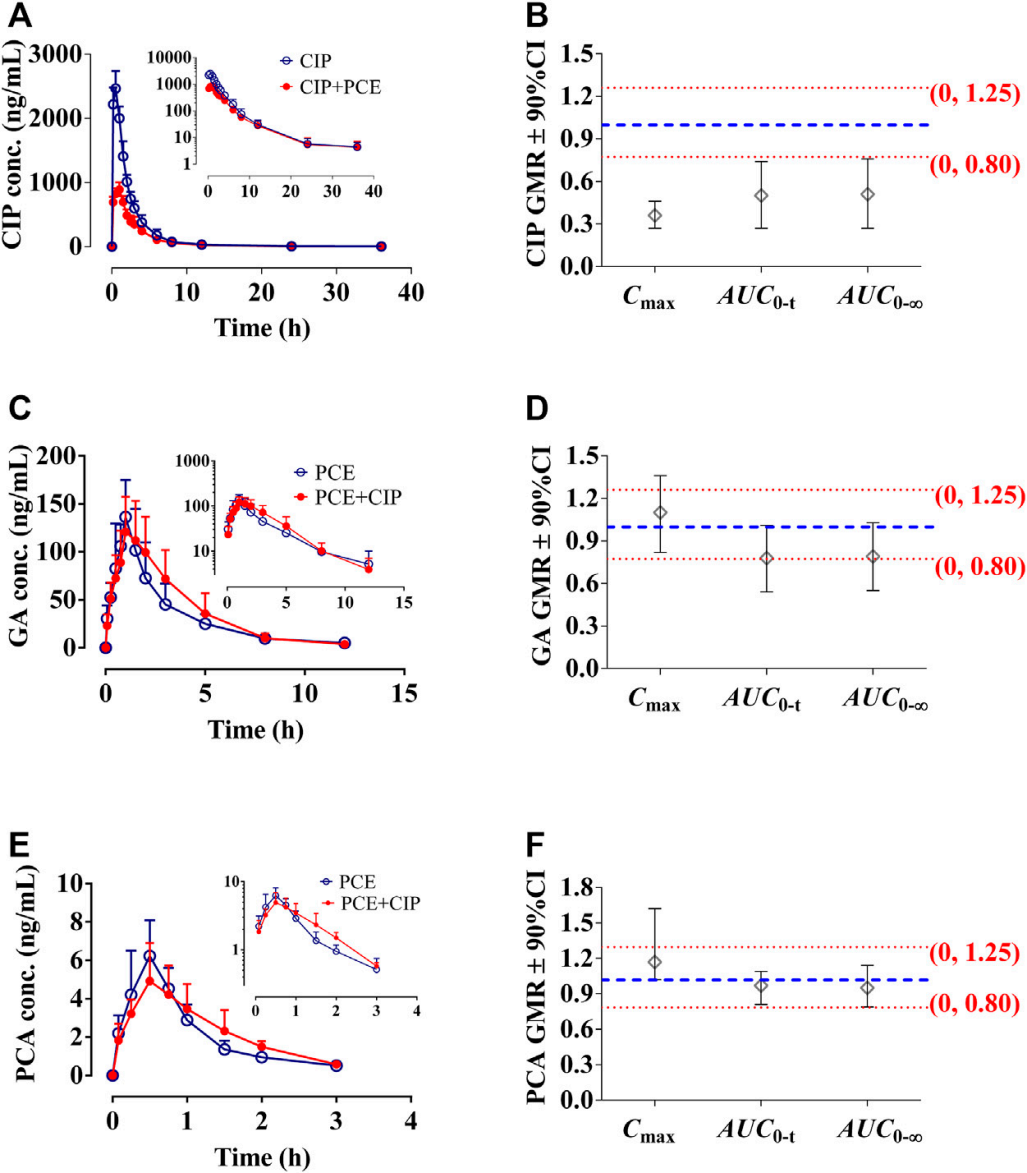
Circulating ciprofloxacin (CIP) concentration-time profiles **(A)** *a*nd the 90% confidence intervals (CI) for the geometric mean ratios [GMR, **(B)**] of *C*_max_, *AUC*_0-t_ and *AUC*_0-∞_ in the presence and absence of PCE to rats, as well as gallic acid [GA, **(C,D)**] and protocatechuic acid [(PCA, **(E,F)**] in the presence and absence of CIP (mean ± SD, *n* = 6).

The effects of PCE on circulating CIP concentrations were observed by comparing changes in PK exposure measures between CIP and CIP + PCE groups. The CIP concentration-time profiles and PK parameters are presented in Figure 5A and Table 3, respectively. The *AUC*_0-t_, *AUC*_0-∞_ and *C*_max_ of circulating CIP were significantly decreased in the presence of single-dose PCE, while the *V*_z_/*F* and *CL*/*F* were significantly increased (Table 3). After combination therapy, the GMRs ±90%CI of ciprofloxacin *AUC*_0-t_ and *C*_max_ were 0.50 (0.27, 0.74) and 0.36 (0.27, 0.46), respectively, falling out of the equivalence range of 0.80–1.25 (Figure 5B). The results indicated that the co-administration of PCE significantly reduces the circulating exposure of CIP in rats.

**TABLE 3:** Pharmacokinetic parameters of ciprofloxacin after intragastric administration of ciprofloxacin (CIP, 0.045 g/kg) with and without PCE (0.72 g/kg) to rats (mean ± SD, *n* = 6).

Parameters	CIP	CIP + PCE
*C*_max_ (ng/ml)	2470 ± 272	892 ± 108*
*AUC*_0-t_ (h·ng/mL)	6090 ± 884	3070 ± 633*
*AUC*_0-∞_ (h·ng/mL)	6120 ± 889	3110 ± 665*
*t*_max_ (h)^a^	0.5 (0.5–0.5)	1.0 (0.5–1.0)*
*k*_e_ (h^−1^)	0.135 ± 0.04	0.143 ± 0.039
*t*_1/2_ (h)	5.49 ± 1.66	5.22 ± 1.84
*V*_z_/F (L)	12.16 ± 5.48	21.40 ± 3.31*
*CL*/F (L/h)	1.50 ± 0.25	2.99 ± 0.60*

^a^ Median (range). *p< 0.05, significant differences were observed when compared with the CIP, group.

#### 3.2.2 Effect of ciprofloxacin on the pharmacokinetics of gallic acid and protocatechuic acid in rats

After combination therapy, the altered circulating exposures of PCE PK-markers (GA and PCA) are shown in Figures 5C–F. The PK parameters of GA and PCA are summarized in Table 4. No significant differences between PCE and PCE + CIP groups were observed on the main PK parameters of GA and PCA. After co-treatment with CIP and PCE, the GMRs ±90% CI of GA *AUC*_0-t_ and *C*_max_ were 0.78 (0.54, 1.01) and 1.10 (0.82, 1.36), respectively (Figure 5D), and the GMRs ± 90% CI of PCA *AUC*_0-t_ and *C*_max_ were 0.97 (0.81, 1.09) and 1.17 (1.02, 1.62), respectively (Figure 5F). The results indicated that the co-treatment with CIP does not significantly change the PK behaviors of GA and PCA in rats, which was the same conclusion drawn from the study in humans.

**TABLE 4:** Pharmacokinetic parameters of gallic acid and protocatechuic acid after intragastric administration of PCE (0.72 g/kg) with and without ciprofloxacin (CIP, 0.045 g/kg) to rats (mean ± SD, *n* = 6).

Parameters	Gallic acid (GA)		Protocatechuic acid (PCA)	
	PCE	PCE + CIP	PCE	PCE + CIP
*C*_max_ (ng/ml)	133.84 ± 36.38	146.75 ± 37.94	5.08 ± 1.82	6.69 ± 1.87
*AUC*_0-t_ (h·ng/mL)	465.36 ± 171.00	363.91 ± 137.00	6.72 ± 2.39	6.37 ± 1.18
*AUC*_0-∞_ (h·ng/mL)	482.79 ± 169.05	384.51 ± 1.49	7.32 ± 2.39	7.04 ± 1.55
*t*_max_ (h)^a^	1.5 (1–1.5)	1 (0.5–1)	0.5 (0.5–0.75)	0.5 (0.25–0.75)
*k*_e_ (h^−1^)	0.35 ± 0.16	0.44 ± 0.26	1.11 ± 0.38	1.10 ± 0.45
*t*_1/2_ (h)	2.62 ± 1.78	1.92 ± 0.74	0.68 ± 0.21	0.76 ± 0.43
*V*_z_/F (L)	8.93 ± 9.83	6.41 ± 1.80	6.50 ± 3.16	6.87 ± 2.54
*CL*/F (L/h)	2.06 ± 0.85	2.58 ± 0.95	6.72 ± 2.19	6.67 ± 1.54

^a^ Median (range). No significant differences were observed when compared with the PCE, group (*p* > 0.05).

### 3.3 Changed tissue distribution of ciprofloxacin and PCE in rats

#### 3.3.1 Effect of PCE on the Tissue Distribution of ciprofloxacin in Rats

**FIGURE 6:**
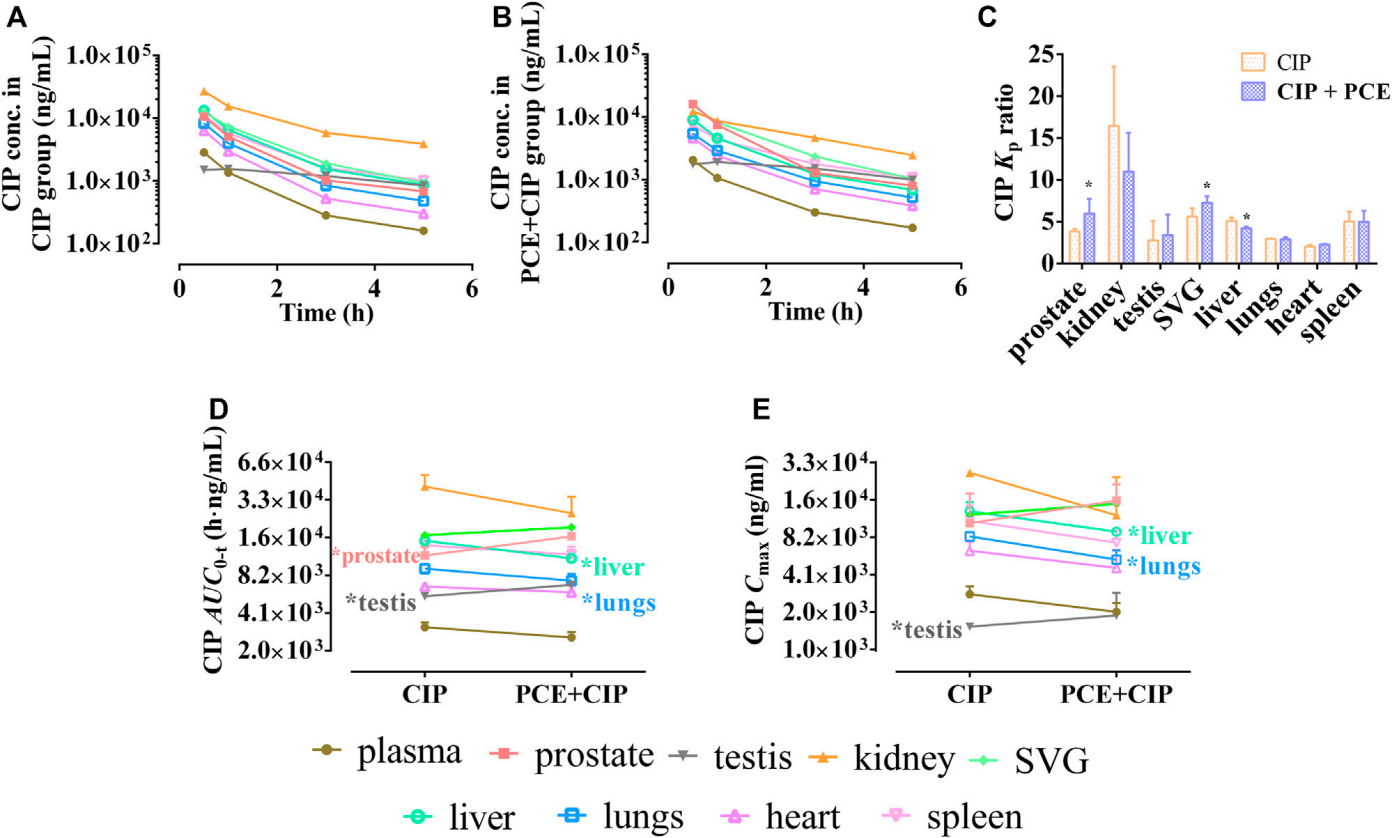
Tissue distribution profiles of ciprofloxacin (CIP) after intravenous administration of CIP in the absence **(A)** and presence **(B)** of PCE to rats, as well as the tissue to plasma distribution coefficients [*K*_p_, **(C)**] and the distribution parameters *AUC*_0-t_ **(D)** and *C*_max_ **(E)** of CIP (mean ± SD, *n* = 3).

PCE had little effect on plasma CIP after intravenous injection using rats (Supplementary Figure S1; Supplementary Table S2). The tissue distribution profiles of CIP in normal rats after intravenous administration of CIP in the absence and presence of PCE are charted in Figures 6A,B, respectively. PCE significantly increased the tissue to plasma distribution coefficients (*K*_p_) of CIP in the prostate and SVG, but decreased its *K*_p_ in the liver (*p* < 0.05, Figure 6C). The *AUC*_0-t_ of CIP in tissues was arranged as follows: kidney > SVG > liver > spleen > prostate > lung in the CIP group, and kidney > SVG > prostate > spleen > liver > lung in the CIP + PCE group (Figure 6D). The *C*_max_ of CIP in tissues was arranged as follows: kidney > liver > SVG > spleen > prostate > lung in the CIP group, and prostate > SVG > kidney > liver > spleen > lung in the CIP + PCE group (Figure 6E). The mean ratios of CIP tissue exposure with and without PCE were more than 1.25 times in the prostate, testis and SVG, but less than 0.8 times in the kidney, liver and lung. PCE significantly increased tissue exposure of CIP in the prostate and testis, but decreased its exposure in the liver and lungs (*p* < 0.05).

#### 3.3.2 Effect of ciprofloxacin on the Tissue Distribution of PCE PK-markers in Rats

**FIGURE 7:**
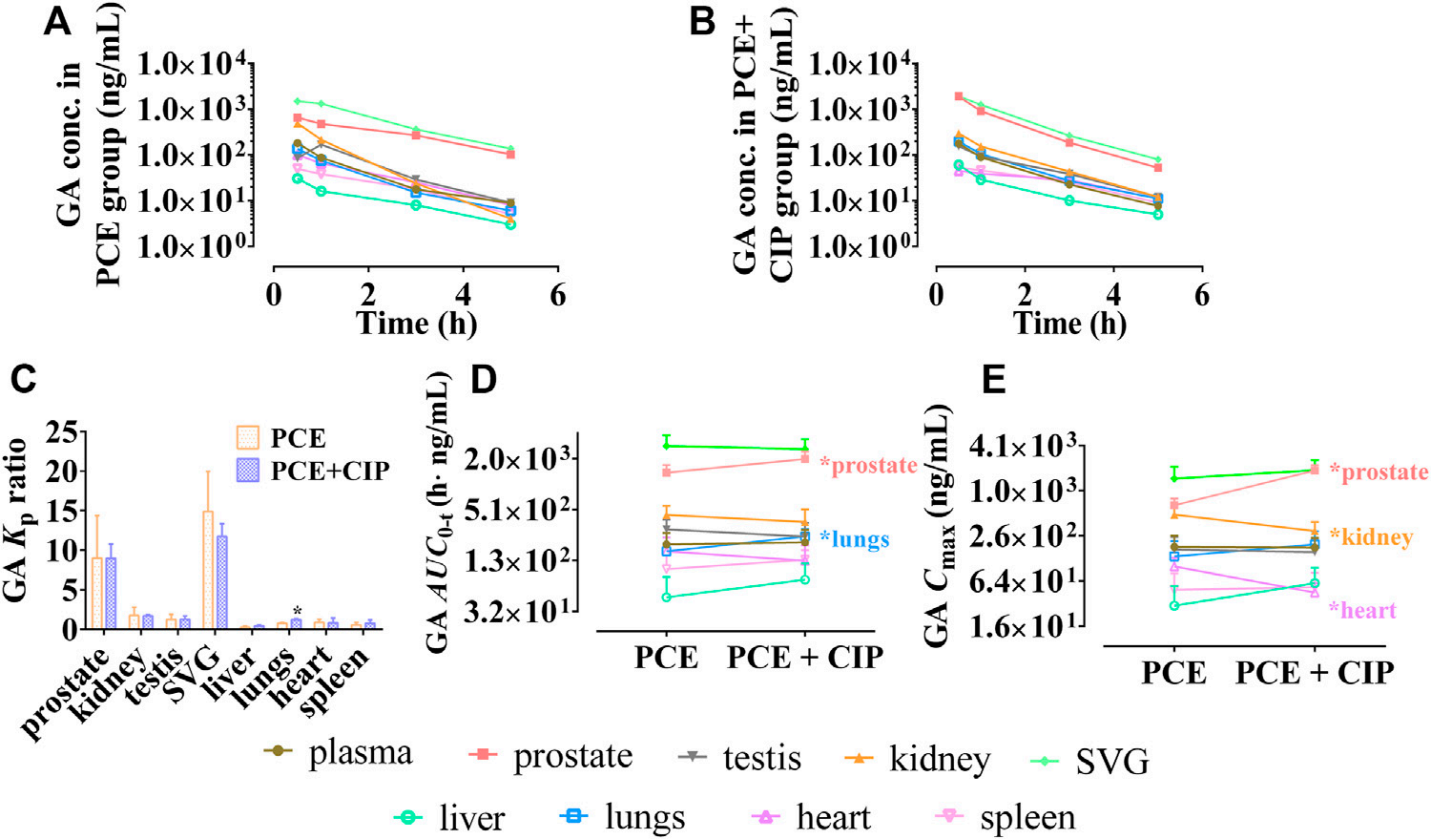
Tissue distribution profiles of gallic acid (GA) after oral administration of PCE in the absence **(A)** and presence **(B)** of ciprofloxacin, as well as the tissue to plasma distribution coefficients [*K*_p_, **(C)**] and the distribution parameters including *AUC*_0-t_ **(D)** and *C*_max_ **(E)** of GA (mean ± SD, *n* = 3).

The tissue distribution curves of GA in normal rats after intragastric administration of PCE in the absence and presence of CIP are displayed in Figures 7A,B, respectively. CIP significantly increased the *K*_p_ of GA in the lungs, but did not significantly alter its *K*_p_ in other tissues (Figure 7C). The *AUC*_0-t_ of GA in tissues was ranked as follows: SVG > prostate > kidney > testis > plasma (Figure 7D). The *C*_max_ of GA in tissues was ranked as follows: SVG > prostate > kidney > plasma > testis in the PCE group, and SVG > prostate > kidney > lung > plasma in the CIP + PCE group (Figure 7E). CIP significantly increased the *AUC*_0-t_ and/or *C*_max_ of GA in the prostate and testis, but decreased its *C*_max_ in the kidney and heart (*p* < 0.05).

**FIGURE 8:**
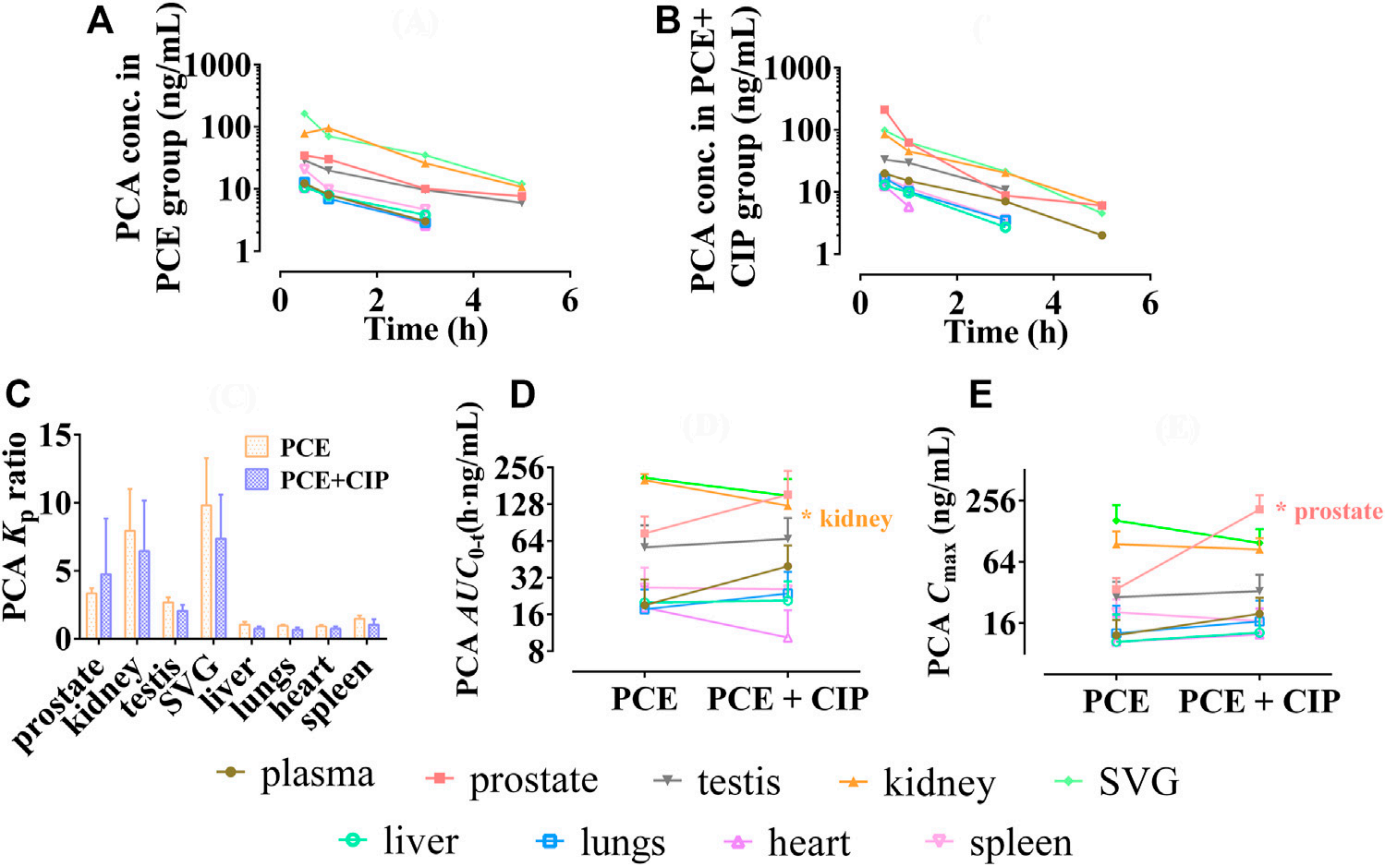
Tissue distribution profiles of protocatechuic acid (PCA) after oral administration of PCE in the absence **(A)** and presence **(B)** of ciprofloxacin, as well as the tissue to plasma distribution coefficients [*K*_p_, **(C)**] and the distribution parameters *AUC*_0-t_ **(D)** and *C*_max_ **(E)** of PCA (mean ± SD, *n* = 3).

The tissue distribution curves of PCA in the absence and| presence of CIP are shown in Figures 8A,B, respectively. CIP did not significantly alter the *K*_p_ of PCA in each tissue (*p* > 0.05, Figure 8C). Both *AUC*_0-t_ and *C*_max_ of PCA in tissues were arranged as follows: SVG > kidney > prostate > testis > spleen in the PCE group, and prostate > SVG > kidney > testis > plasma in the CIP + PCE group (Figures 8D,E). CIP significantly increased the *C*_max_ of PCA in the prostate, but decreased its *AUC*_0-t_ in the kidney (*p* < 0.05).

### 3.4 Effect of PCE on the transport of ciprofloxacin mediated by multiple transporters

#### 3.4.1 Inhibitory effects of gallic acid and protocatechuic acid on the activity of multiple transporters

**FIGURE 9:**
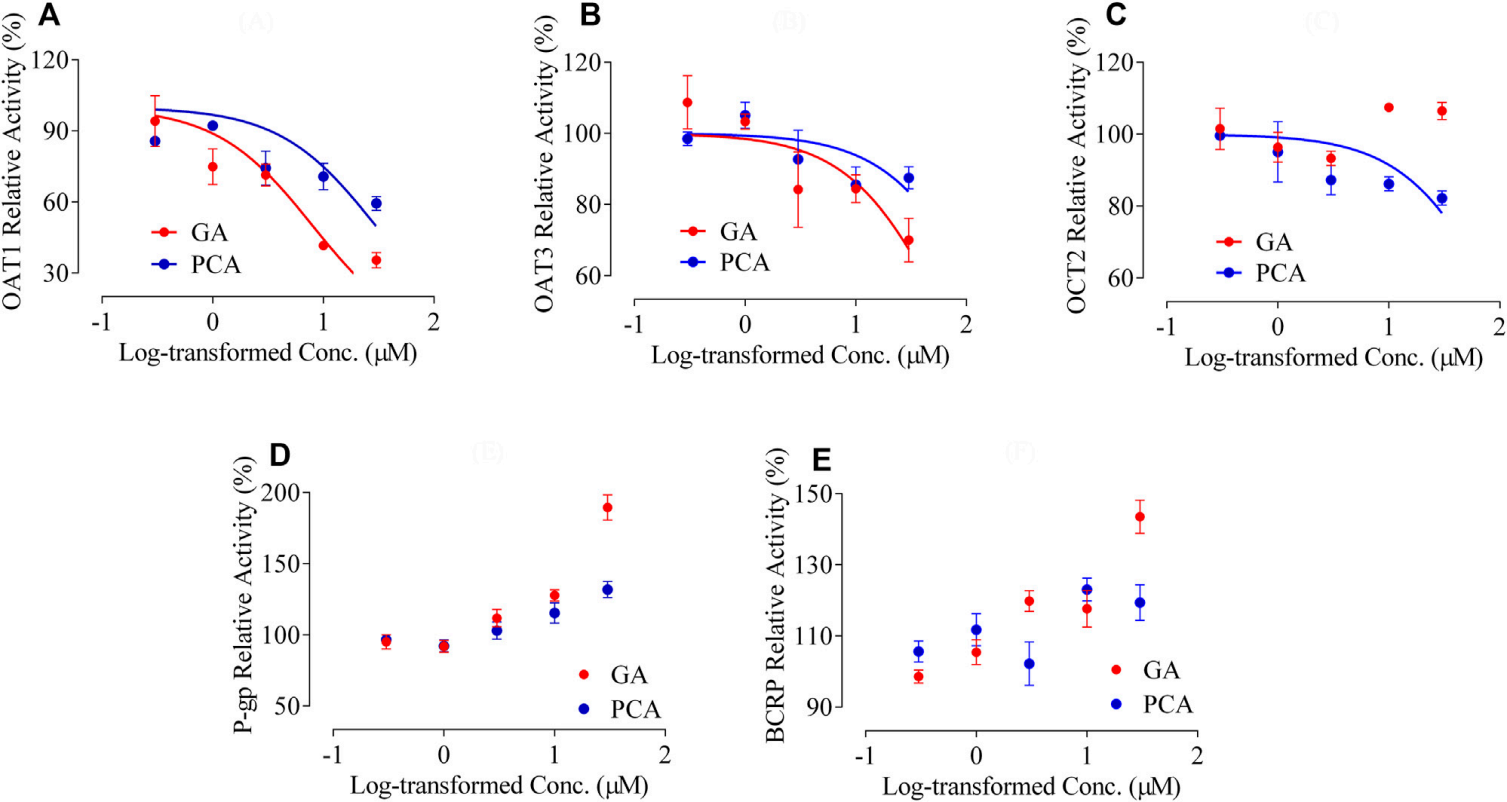
Inhibitory effects of GA and PCA on the activity of multiple transporters, including human organic anion transporter 1 [hOAT1, **(A)**], [hOAT3 **(B)**], human organic cation transporter 2 [hOCT2, **(C)**], human multidrug resistance protein 1 [hMDR1, **(D)**], and human breast cancer resistance protein [hBCRP, **(E)**] (mean ± SD, *n* = 3).

Significant inhibitory effects of the positive inhibitors of probenecid, cimetidine, rifampicin, verapamil, and cyclosporine were presented on the transporters hOAT1, hOAT3, hOCT2, hMDR1, and hBCRP, respectively (Supplementary Figure S2). A concentration-dependent inhibition type was observed on the transporters hOAT1 and hOAT3 for GA, as well as the transporters hOAT1, hOAT3 and hOCT2 for PCA (Figures 9A–C). GA and PCA presented significant inhibitory effects on the hOAT1-mediated uptake of ^14^C-PAH with the IC_50_ values 8.01 and 29.73 µM, respectively (Figure 9A). Weak or ineffective inhibitions were observed on hOAT3 for GA (IC_50_ = 62.33 µM) and PCA (IC_50_ = 152.4 µM, Figure 9B), as well as hOCT2 for PCA (IC_50_ = 107.0 µM, Figure 9C). Meanwhile, no inhibitory effect was observed on MDR1 or BCRP′ activity after co-incubation with GA and PCA. Interestingly, it shows a concentration-dependent increase in the efflux of MDR1-mediated rhodamine 123 or BCRP-mediated lucifer yellow with the increased GA and PCA’s concentration ranges from 0.3 to 30 µM (Figures 9D,E).

#### 3.4.2 Effect of PCE on the bidirectional transport of ciprofloxacin in Caco-2 cells

**FIGURE 10:**
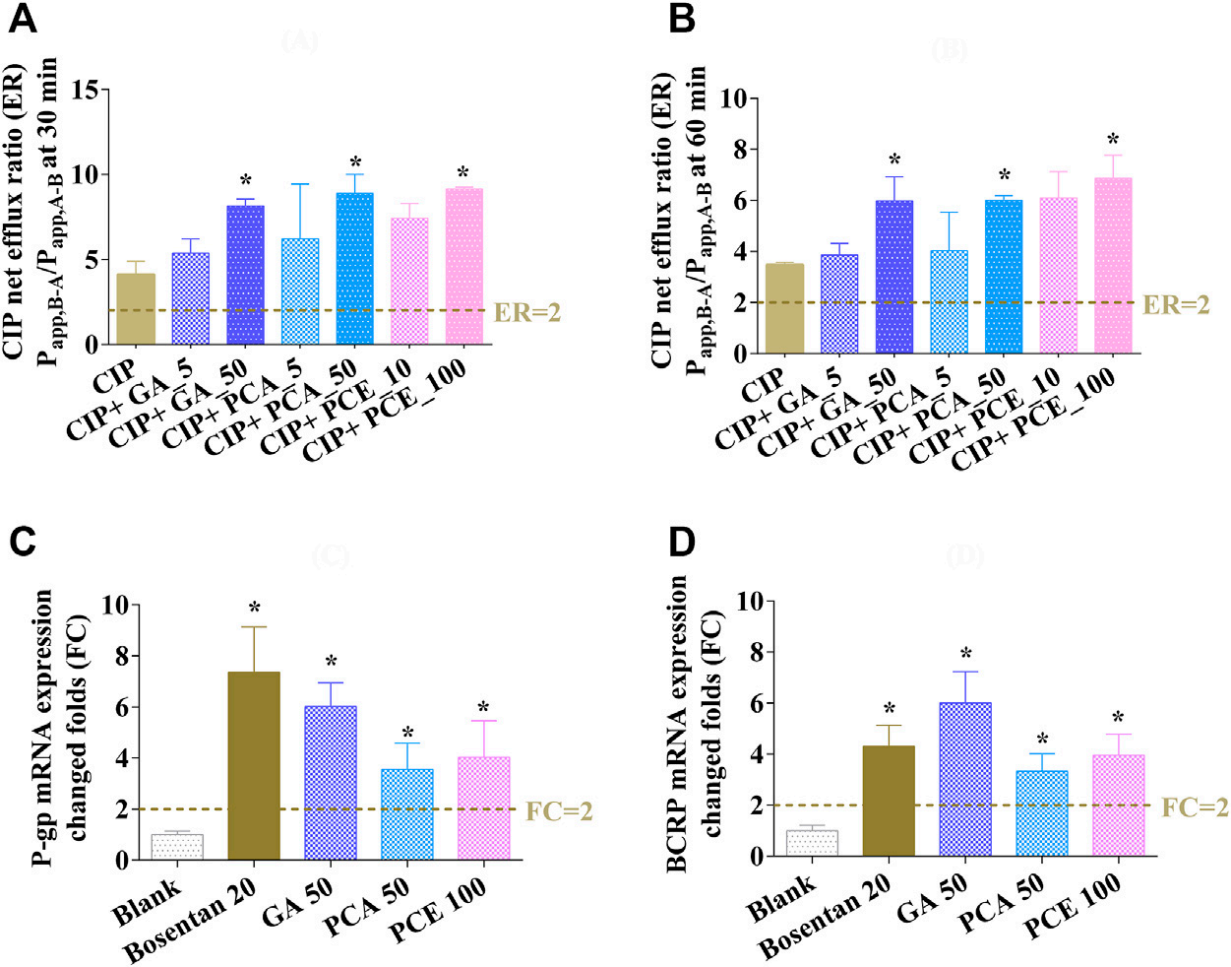
Effects of gallic acid (GA), protocatechuic acid (PCA) and PCE on the change of ciprofloxacin efflux ratios at 0.5 h **(A)** and 1 h **(B)**, and the mRNA expressions of MDR1 **(C)** and BCRP **(D)** in Caco-2 cells (mean ± SD, *n* = 3).

In the bidirectional transport assay with Caco-2 cells, the net flux ratio of CIP after 0.5 and 1 h were 4.13 ± 0.76 and 3.48 ± 0.10, respectively. After co-incubation with GA, PCA and PCE, the net flux ratio of CIP showed varying degrees of enlargement (Figures 10A,B). A significant improvement was observed in the net efflux ratios after co-treatment with GA (50 μM), PCA (50 μM), and PCE (100 mg/ml) (*p* < 0.05). The increased net flux ratio facilitates the flow of CIP from the blood to the intestinal lumen, renal tubules or bile ducts, which may be related to the reduction of CIP systemic exposure.

#### 3.4.3 Effect of PCE on the expressions of MDR1 and BCRP in Caco-2 cells

The effect of PCE on the expression of MDR1 and BCRP mRNAs are shown in Figures 10C,D, respectively. After pretreatment with bosentan, the expression of P-gp and BCRP mRNAs in Caco-2 cells were increased by 7.36- and 4.31-fold, respectively. After pretreatment with the investigated drugs, the expression of MDR1 and BCRP mRNAs increased 6.02- and 6.00-fold in cells treated with GA, 3.56- and 3.33-fold in cells treated with PCA, and 4.04- and 3.95-fold in cells treated with PCE, respectively. The increased MDR1 and BCRP mRNA expressions may contribute to CIP efflux from the blood circulatory system.

## 4 Discussion

Herbal medicinal products are commonly used as a complementary or alternative treatment for a variety of diseases, rehabilitation and health care (Meng and Liu, 2014). Thereafter, due to the pharmacokinetic- and pharmacodynamic-based HDIs, the concurrent use of herbal medicinal products may mimic, magnify, or oppose the effects of medicinal products (Fugh-Berman, 2000). Pharmacokinetic HDIs may increase or decrease the systemic exposures of either component through multiple mechanisms, including drug-metabolizing enzymes (*e.g.*, CYP450s and UGTs), drug transporters (*e.g.*, P-gp and BCRP) and plasma protein (*e.g.*, albumin and globulin), which is basically similar to pharmacokinetic DDIs (Li et al., 2019). Nevertheless, the research of pharmacokinetic HDIs is generally more challenging than that of DDIs, given the complicated herbal components and the batch-to-batch variation of herbal medicines (Meng and Liu, 2014). This has led to a phenomenon that most of the current studies pay more attention to the unidirectional effect (herbs→drugs) than the bidirectional effect (herbs↔drugs). Even though, the pharmacokinetic HDIs between PCE and CIP were investigated on a bidirectional effect (PCE↔CIP) in this study. GA and PCA were identified as appropriate PK-marker components of *P. capitatum* because of 1) their extensive pharmacological activities consistent with *P. capitatum* (Liao et al., 2011; Khan et al., 2015; Choubey et al., 2018; Song et al., 2020; Bai et al., 2021), 2) their rich *in vitro* (Liao et al., 2013; Zhang et al., 2013a; Zhang et al., 2013b; Li et al., 2021b) and *in vivo* exposures (Ma et al., 2015, 2016; Huang et al., 2019; Li et al., 2021b; Guan et al., 2022), and 3) their acceptable pharmacokinetic properties (Ma et al., 2015; Li et al., 2021b) and targeted distribution in kidney tissue (Ma et al., 2016). However, there is a risk that the two compounds were possibly not the most critical compounds responsible for PCE’s effect, and the use of any single compound of the two compounds in the study could not reflect the overall effect of PCE.

Since CIP is a substrate of multiple transporters, the drug-transporter mediated inhibition test was performed to study the responsibilities for the reduced CIP exposure. Although herbal crude extract has been used as perpetrator in some cell experiments, false positive or false negative results will inevitably occur (Ge et al., 2010). The main reasons include but are not limited to the following aspects. PCE addition can alter some extracellular characteristics, such as pH value and ionic strength. PCE contains some components that are not absorbed into blood when administered orally, which may interfere with cultured cells. Despite serum pharmacological method can solve the above problems, preparation of test serum for cell experiment is a complicated process: Besides chemicals or heat pretreatment, it involves the proteolytic cascades of coagulation along with complement, fibrinolysis and kinin systems, as well as leukocyte and platelet activation resulting in release reactions (Ge et al., 2010). The pretreatment deviates serum sample elements away from the original *in vivo* state. The results obtained from the drug-containing serum are at least partially uncertain in its validity. Considering GA and PCA are highly exposed in plasma and urogenital system tissues, we examined the inhibitory effects of the two components on the activity of multiple transporters that mediate CIP transport. The shortcomings of using these two compounds are the same as those of using PK-markers.

Clinical evidence showed that combination of PCE and CIP could produce a better effect for the treatment of chronic prostatitis (Zhou et al., 2016) and urinary tract infections (Pu et al., 2016). However, since co-administration of PCE significantly reduces plasma CIP in human (Figure 2), the results seemed not to support such an effect. Interestingly, we found that the combined therapy significantly increased the exposure of CIP in the prostate (Figure 6), which may be the target tissue for the treatment of chronic prostatitis (Lipsky et al., 2010). In addition to CIP, GA and PCA (PCE PK-markers) presented high exposure in the urogenital system, such as prostate, kidney, and seminal vesicle gland (Figures 7, 8). The antibacterial, anti-inflammatory, antioxidant and analgesic activities of PCE, GA and PCA are helpful to improve the efficacy of CIP in the treatment of infectious diseases of urogenital system (Liao et al., 2011; Khan et al., 2015; Choubey et al., 2018; Song et al., 2020; Bai et al., 2021). The *in vitro* study proves that PCA increased up to 50% of the antibacterial activity, especially that of levofloxacin against *Staphylococcus aureus* and *Escherichia coli* (Fifere et al., 2022).

Concurrent use of PCE significantly reduced circulating CIP in humans and rats, consistent with previous reports (Lu et al., 2016). Considering the manner in which the drug is removed from the body, CIP is primarily cleared by active tubular secretion (up to 2/3 of the total clearance, Höffken et al., 1985; Mulgaonkar et al., 2012) and intestinal excretion (approximately 18%, Rohwedder et al., 1990). Since the liver metabolism (approximately 10%) and biliary excretion (approximately 1%) of the drug is relatively low (Vance-Bryan et al., 1990), the reduced circulating CIP in humans was mainly attributed to the change of CIP across the renal and intestinal epithelia. CIP is a known substrate of the ATP-binding cassette transporters BCRP (Alvarez et al., 2008; Haslam et al., 2011) and MDR1 (Brillault et al., 2010; Arakawa et al., 2012; Zhang et al., 2019; Zimmermann et al., 2019), both of which are located in the apical (luminal) membrane of kidney proximal tubules and intestinal epithelia (International Transporter Consortium, 2010). The reduced circulating CIP in humans may be attributed to the increased BCRP/MDR1-mediated CIP efflux from blood to feces and urine, as indicated by the effects of PCE, GA and PCA on CIP bidirectional transport and BCRP/MDR1 expressions in this study. Due to the zwitterionic nature, CIP is likely to interact with organic anion and cation transporters, such as OAT1, OAT3, and OCT2 (Dautrey et al., 1999; Vanwert et al., 2008; Arakawa et al., 2012; Mulgaonkar et al., 2012; Ong et al., 2013). But the inhibitory effects of GA and PCA on OAT1/3-mediated CIP secretion from blood to feces and urine did not contribute to the reduced circulating CIP in humans. That is, the decrease of CIP systematic exposure in the combined group was caused by the integrated effect of synergy/antagonism among multiple transporters. Additionally, the reduced circulating CIP may also come from other transporter-mediated interactions (eg., OATPs, MRPs, Wu et al., 2012; Marquez et al., 2009), drug metabolizing enzyme-mediated interactions (eg., CYP450, Zheng et al., 2014), and other PCE composition-perpetrated interactions (eg., flavonoids, An et al., 2014).

Tissue distribution-based HDI studies were also implemented to assess the effects of combination therapy on the altered distribution kinetics of CIP, GA, and PCA, which may directly or indirectly lead to changes in their therapeutic effect. The combined administration increased CIP exposure in the prostate, testis, and seminal vesicle gland, which may help to treat infectious diseases of the male reproductive system. As summarized by Obligacion et al. (2006), the human prostate tissue contains the multidrug resistance protein (MRP) transporters MRP1, MRP2, MRP3, and MRP4, and MDR1. Thus, the increased distribution of CIP in the prostate gland may be attributed to the activation of MRPs and MDR1 after treated with GA and PCA. Although no significant alteration in the circulating GA and PCA after combination therapy, there were some elevated distribution kinetics and tissue exposures in the prostate and lung. Interestingly, the exposures of CIP, GA, and PCA all showed an increase in the prostate tissue after combined administration, which will contribute to the treatment of chronic prostatitis. A meta-analysis of randomized controlled trials of combination therapy indicated that PCE-based products (Relinqing^®^) combined with fluoroquinolones can improve the total effective rate of chronic prostatitis compared with fluoroquinolones alone (Zhou et al., 2016). The accumulation of CIP and PCE PK-marker components in prostate tissue is helpful to explain the above clinical phenomenon.

In summary, we focused on the pharmacokinetic herb-drug interactions between PCE and CIP in humans, rats and cells. The effects of PCE↔CIP presented significantly reduced plasma exposure of CIP and almost unchanged exposure of PCE PK-markers (GA and PCA) in humans and rats. The reduced plasma CIP may be attributed to integrated routes mediated by multiple transporters, including BCRP, MDR1, and OAT1/3. GA and PCA were highly exposed to urogenital tissues after intragastric administration of PCE with or without CIP to rats. Although the decreased circulating CIP seemed not to support the clinical synergism of CIP and PCE, the combined therapy increased the exposure of CIP, GA and PCA in the prostate gland. The enrichment of these drugs in prostate will be helpful to the treatment of chronic bacterial prostatitis. Additionally, the effect of combination therapy should pay attention to both the pharmacokinetic- and pharmacodynamic-based interactions, rather than unilaterally. Further in-depth research should be carried out in the future.

## Data Availability

The original contributions presented in the study are included in the article/Supplementary Material, further inquiries can be directed to the corresponding author.
