# Supplementary material for: Pharmacokinetic herb-drug interactions: Altered systemic exposure and tissue distribution of ciprofloxacin, a substrate of multiple transporters, after combined treatment with Polygonum capitatum Buch.-Ham. ex D. Don extracts
Source: Front Pharmacol. 2022 Oct 25;13:1033667. doi: 10.3389/fphar.2022.1033667 (PMC9640990; doi:10.3389/fphar.2022.1033667)
Supplement: Supplementary file 1 [file Table1.doc]

**Table S1.** Demographic characteristics of healthy subjects in the herb–drug interaction study (n=12).

| Characteristic | Mean ± SD (range) |
| --- | --- |
| Subject number | 12 |
| Male/female | 12/0 |
| Age (years) | 27.8±3.57 (20~32) |
| Height (m) | 1.70±0.07 (1.53~1.78) |
| Weight (kg) | 66.6±6.94 (54.3~76.6) |
| BMI (kg/m^2^) | 23.1±2.13 (20.0~25.7) |
