# Supplementary material for: Pharmacokinetic herb-drug interactions: Altered systemic exposure and tissue distribution of ciprofloxacin, a substrate of multiple transporters, after combined treatment with Polygonum capitatum Buch.-Ham. ex D. Don extracts
Source: Front Pharmacol. 2022 Oct 25;13:1033667. doi: 10.3389/fphar.2022.1033667 (PMC9640990; doi:10.3389/fphar.2022.1033667)
Supplement: Supplementary file 2 [file Table2.docx]

**Supplementary Table S2**. Estimated pharmacokinetic parameters of ciprofloxacin after intravenous administration of ciprofloxacin (CIP, 0.036 g/kg) with and without PCE (0.72 g/kg) to rats (mean ± SD, n=3).

| Parameters | CIP | CIP + PCE |
| --- | --- | --- |
| *k*_e_ (h^-1^) | 0.63±0.05 | 0.52±0.06 |
| *t*_1/2_ (h) | 1.11±0.08 | 1.36±0.18 |
| *AUC*_0-t_ (h·ng/mL) | 5349.98±686.08 | 4717.62±854.38 |
| *AUC*_0-∞_ (h·ng/mL) | 5608.81±661.80 | 5060.68±995.00 |
| *V*_z_ (L) | 2.61±0.54 | 3.01±0.39 |
| *CL*(L/h) | 1.62±0.21 | 2.08±0.42 |

No significant differences were observed when compared with the CIP group (*p* > 0.05).
