# Supplementary material for: Pharmacokinetic herb-drug interactions: Altered systemic exposure and tissue distribution of ciprofloxacin, a substrate of multiple transporters, after combined treatment with Polygonum capitatum Buch.-Ham. ex D. Don extracts
Source: Front Pharmacol. 2022 Oct 25;13:1033667. doi: 10.3389/fphar.2022.1033667 (PMC9640990; doi:10.3389/fphar.2022.1033667)

**Supplementary Figure S2**. Effect of positive inhibitors on the transporters of hOAT1, hOAT3, hOCT2, hMDR1, and hBCRP in the hOAT1-MDCK, hOAT3-MDCK, hOCT2-S2, hMDR1-MDCK, and hBCRP-MDCK cells, respectively (Mean ± SD, n=3).


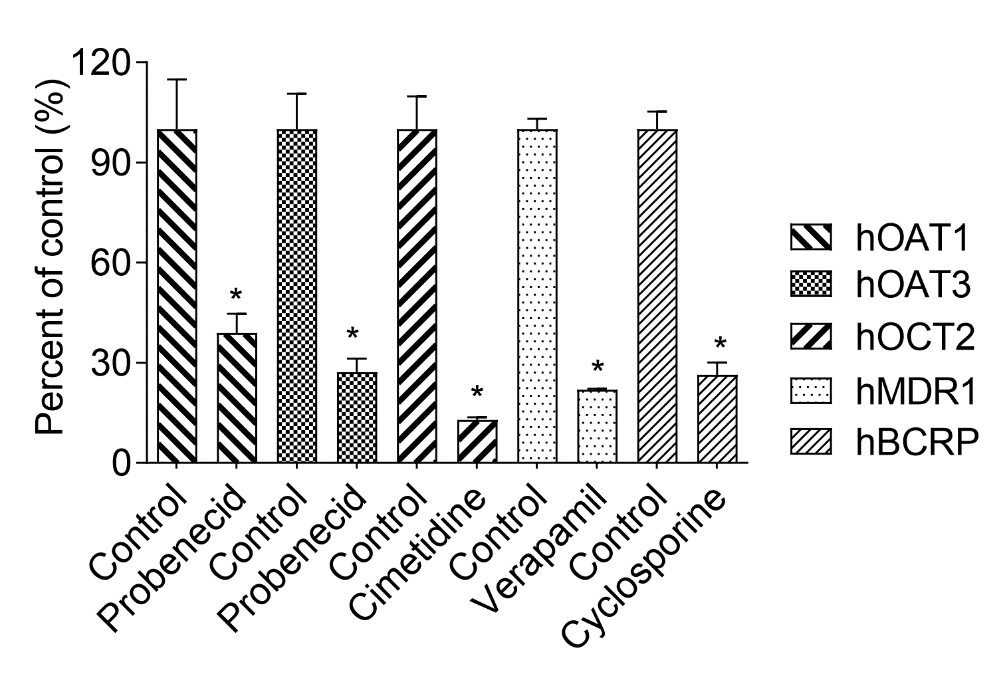

Supplement: Supplementary file 3 [file DataSheet2.docx]
