# Supplementary material for: Pharmacokinetic herb-drug interactions: Altered systemic exposure and tissue distribution of ciprofloxacin, a substrate of multiple transporters, after combined treatment with Polygonum capitatum Buch.-Ham. ex D. Don extracts
Source: Front Pharmacol. 2022 Oct 25;13:1033667. doi: 10.3389/fphar.2022.1033667 (PMC9640990; doi:10.3389/fphar.2022.1033667)
Supplement: Supplementary file 4 [file DataSheet1.docx]

**Supplementary Figure S1**. Plasma ciprofloxacin (CIP) profiles after intravenous administration of CIP in the absence and presence of PCE to rats (mean ± SD, n=3).
